# Supplementary material for: Comparative Analysis of PGRP Family in Polymorphic Worker Castes of Solenopsis invicta
Source: Int J Mol Sci. 2024 Nov 15;25(22):12289. doi: 10.3390/ijms252212289 (PMC11594682; doi:10.3390/ijms252212289)
Supplement: Supplementary file 1 [file ijms-25-12289-s001.zip › ijms-3296925-supplementary.pdf]

Supplementary Material

Table S1. GenBank accession numbers of the genes utilized to construct phylogenetic trees

Name	ID	GenBank Accession Number
Homo sapiens	*HsPGRP-1*	NP_005082.1
	HsPGRP-2	NP_443122.3
	HsPGRP-3	XP_011507420.1
	HsPGRP-4	XP_011508094.1
Drosophila melanogaster	*DmPGRP-SA*	NP_001285128.1
	DmPGRP-LE	NP_001245695.1
	DmPGRP-LC	NP_001246693.1
	DmPGRP-LF	NP_648299.3
	DmPGRP-LD	NP_001137893.1
	DmPGRP-SD	NP_648145.1
	DmPGRP-LA	NP_996026.1
	DmPGRP-SB1	NP_648917.1
	DmPGRP-SB2	NP_001261970.1
	DmPGRP-LB	NP_001247052.1
	DmPGRP-SC2	NP_610410.1
	DmPGRP-SC1a	NP_610407.1
	DmPGRP-SC1b	NP_001286209.1
Schistocerca gregaria	*SgPGRP-SA*	XP_049828074.1
	SgPGRP-SD	XP_049828931.1
	SgPGRP-LA	XP_049864618.1
	SgPGRP-LB	XP_049839353.1
	SgPGRP-LC	XP_049839352.1
	SgPGRP-SC2	XP_049860300.1
	SgPGRP-1	XP_049860522.1
Zootermopsis nevadensis	*ZnPGRP-2*	XP_021922592.1
	ZnPGRP-LC	XP_021932118.1
	ZnPGRP-LA	XP_021921577.1
	ZnPGRP-SB1	XP_021940814.1
	ZnPGRP-LB	XP_021940816.1
	ZnPGRP-SC2	XP_021940116.1
Cryptotermes secundus	*CsPGRP-2*	XP_023723966.1
	CsPGRP-LC	XP_023717812.1
	CsPGRP-SD	XP_023720071.1
	CsPGRP-LA	XP_023708935.1
	CsPGRP-SB1	XP_023716715.1
	CsPGRP-LB	XP_023716716.1
	CsPGRP-SC2	XP_033606966.1
Anopheles gambiae	*AgPGRP-S1*	XP_310547.4
	AgPGRP-LC2	XP_558599.3
	AgPGRP-LC3	XP_558600.3

	AgPGRP-LC1	XP_314103.4
	AgPGRP-LB	XP_003435776.1
Sitophilus oryzae	*SoPGRP-1*	XP_030760411.1
	SoPGRP-LA	XP_030762760.1
	SoPGRP-LB	XP_030748448.1
Tenebrio molitor	*TmPGRP-SA*	BAE78510.1
	TmPGRP-SB	CCV65021.1
	TcPGRP-SA	XP_008192927.1
	TcPGRP-LE	XP_968926.1
Tribolium castaneum	*TcPGRP-LD*	XP_970847.1
	TcPGRP-LA	XP_008192537.1
	TcPGRP-LB	XP_969556.1
	BmPGRP-S2	XP_028027285.1
	BmPGRP-S1	XP_028043866.1
	BmPGRP-L6	XP_004929966.1
	BmPGRP-L4	XP_012549076.1
	BmPGRP-L2	XP_004929814.1
Bombyx mandarina	*BmPGRP-L5*	XP_004929948.1
	BmPGRP-L3	XP_037866574.1
	BmPGRP-L1	XP_004929813.1
	BmPGRP-S4	XP_021205511.1
	BmPGRP-S3	NP_001243949.1
	BmPGRP-S5	XP_028035267.1
	BmPGRP-S6	XP_012548099.1
	ObPGRP-S2	XP_019886201.1
Ooceraea biroi	*ObPGRP-LE*	XP_026828474.1
	ObPGRP-LC	XP_019887088.2
	ObPGRP-S2	XP_019886201.1
Monomorium pharaonis	*MpPGRP-S*	XP_012541593.1
	MpPGRP-LC	XP_012532094.1
Bombus terrestris	*BtPGRP-S2*	XP_048269711.1
	BtPGRP-LC	XP_020719583.1
	AmPGRP-S3	NP_001157187.1
Apis mellifera	*AmPGRP-S2*	NP_001157188.1
	AmPGRP-S1	XP_001121036.2
	AmPGRP-SA	AGM19449.1
	AdPGRP-SA	XP_006624068.1
Apis dorsata	*AdPGRP-LA*	XP_006618726.1
	AdPGRP-SC2	XP_031365722.1
	AdPGRP-S1	XP_031365728.1
	AcPGRP-S2	XP_061934990.1
Apis cerana	*AcPGRP-LC*	XP_016914774.1
	AcPGRP-SC2	XP_016922186.1
	AcPGRP-S1	XP_016922185.2

	OtPGRP-LE	XP_022914682.1
Onthophagus taurus	*OtPGRP-2*	XP_022903161.1
	OtPGRP-LF	XP_022914149.1
	MsPGRP-SC	XP_037299857.1
Manduca sexta	*MsPGRP-SB2*	XP_030031132.2
	MsPGRP-3	XP_030021983.2
	MsPGRP-LB	XP_037292723.1
	NvPGRP-SC2	XP_017776602.1
Nicrophorus vespilloides	*NvPGRP-LC*	XP_017776604.1
	NvPGRP-LA	XP_017776594.1
Agrilus planipennis	*ApPGRP-LA*	XP_025830017.1
Bactrocera oleae	*BoPGRP-SB1*	XP_014099773.1
	BoPGRP-SC2	XP_014085196.1

Table S2. PCR primers.

Gene name	Gene number	Primer name	Product length(bp)	Primer sequences（5’-----3’）
SiPGRP-S1	LOC105196159	PGRP S1-F	245	CCAGGAGACTACTGCCAACTTG
		PGRP S1-R		TTCCGTCTTCGCCGACTAGG
SiPGRP-S2	LOC105200397	PGRP S2-F	181	CAGCATACGACCACCGACAAATG
		PGRP S2-R		CGTACGTGTGTGCTCCTTCGC
SiPGRP-S3	LOC105203375	PGRPS3-F	200	TGTTGGCACTTCCTGCTAGT
		PGRPS3-R		ATGGTGTCTTCGAACGCTGT
SiPGRP-L	LOC105197895	PGRP L-F	248	CCTTGTCGGAGGCGATGGTCG
		PGRP L-R		CAGGACTGAGAGTCCCTGGCG
RPL18 (reference gene)	LOC105198518	RPL18F	165	TTTACGGCTCCTCGTCAAGC
		RPL18R		ACGCAATTCTCCCTTCCAGG

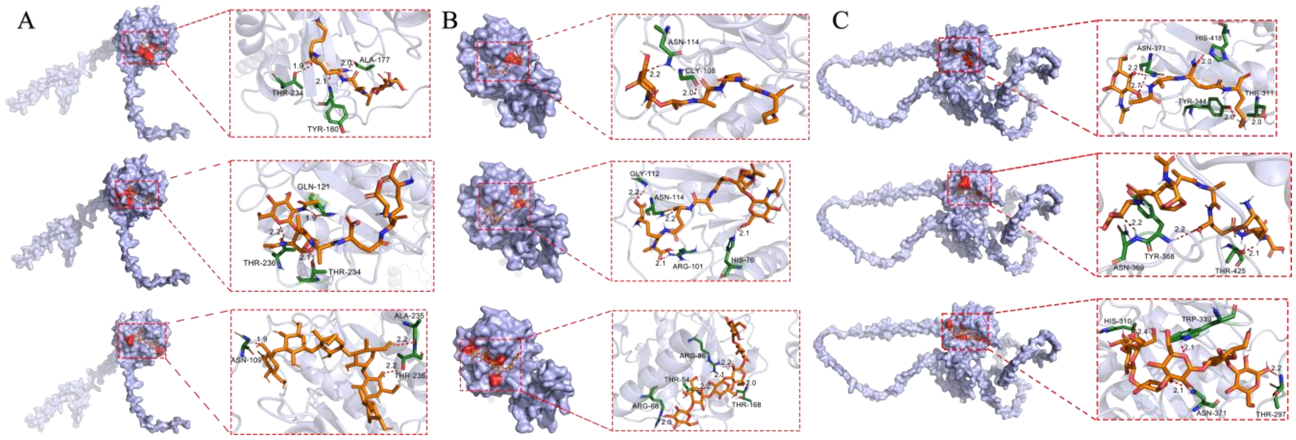

Figure S1. The docking view illustrates the binding interactions of *SiPGRPs* with various ligands specifically TCT (tracheal cytotoxin), MTP (muramyl tripeptide), and laminarihexaose arranged from top to bottom. Panels (A), (B), and (C) depict SiPGRP-S2, SiPGRP-S3, and SiPGRP-L, respectively.

Hydrogen bonds are indicated by red dashed lines, while the ligands are represently as orange sticks. The interacting residues of the receptor are illustrated as green sticks.

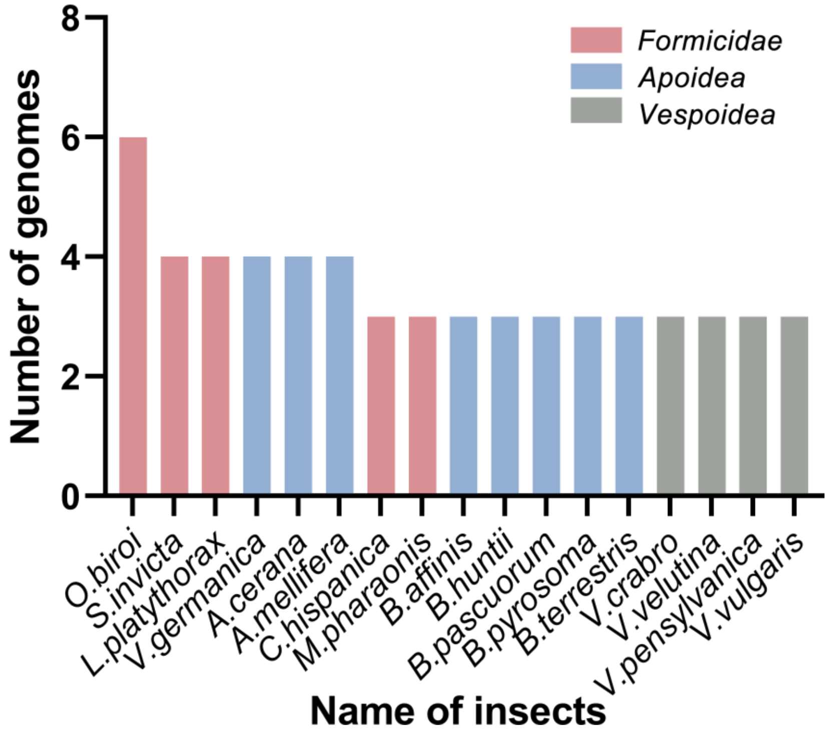

Figure S2. Number of Peptidoglycan Recognition Proteins (PGRPs) identified in the chromosome-level genome assembly of social insects identified in the chromosome-level genome assembly of social insects within the *Hymenoptera* order. The GenBank accession numbers for the respective insect species are as follows: *Ooceraea biroi (O. biroi)*, GCF_003672135.1; *Lasius platythorax* (*L. platythorax*), GCA_964030505.2; *Vespula germanica* (*V. germanica*), GCA_014466195.1; *Apis cerana* (*A. cerana*), GCF_029169275.1; Apis mellifera (A. mellifera), GCF_003254395.2; *Bombus affinis* (*B. affinis*), GCF_024516045.1; *Bombus huntii* (*B. huntii*), GCF_024542735.1; *Bombus pascuorum* (*B. pascuorum*), GCF_905332965.1; *Bombus pyrosoma* (*B. pyrosoma*), GCF_014825855.1; *Bombus terrestris* (*B. terrestris*), GCF_910591885.1; *Cataglyphis hispanica* (*C. hispanica*), GCF_021464435.1; *Monomorium pharaonis* (*M. pharaonis*), GCF_013373865.1; *Vespula crabro* (*V. crabro*), GCF_910589235.1; *Vespula velutina* (*V. velutina*), GCF_912470025.1; *Vespula pensylvanica* (*V. pensylvanica*), GCF_014466175.1; and *Vespula vulgaris* (*V. vulgaris*), GCF_905475345.1.
